# Determining the cost and cost-effectiveness of childhood cancer treatment in Haiti

**DOI:** 10.3332/ecancer.2024.1675

**Published:** 2024-02-28

**Authors:** Nancy S Bolous, Peter Mercredi, Miguel Bonilla, Paola Friedrich, Nickhill Bhakta, Monika L Metzger, Pascale Y Gassant

**Affiliations:** 1 Department of Global Pediatric Medicine, St Jude Children’s Research Hospital, Memphis, TN 38105, USA; 2 Independant Researcher; 3 Médecins Sans Frontières, Geneva 1202, Switzerland; 4 Nos Petit Frères et Sœurs-St Damien Hospital, Port-au-Prince 6124, Haiti

**Keywords:** cost-effectiveness, cost, children, paediatric, cancer, Haiti

## Abstract

Haiti is a low-income country with one of the lowest human development index rankings in the world. Its childhood cancer services are provided by a single hospital with the only dedicated paediatric oncology department in the country. Our objective was to assess the cost and cost-effectiveness of all types of childhood cancer in Haiti to help prioritise investments and to support national cancer control planning. All costing data were collected from the year 2017 or 2018 hospital records. Costs were classified into 11 cost categories, and the proportion of the overall budget represented by each was calculated and converted from Haitian Gourde to United States dollars. The 5-year survival rate was retrieved from hospital records and used to calculate the cost-effectiveness of disability-adjusted life year (DALY) averted, using a healthcare costing perspective. Additional sensitivity analyses were conducted accounting for late-effect morbidity and early mortality and discounting rates of 0%, 3% and 6%. The annual cost of operating a paediatric oncology unit in Haiti treating 74 patients with newly diagnosed cancer was $803,184 overall or $10,854 per patient. The largest cost category was pharmacy, constituting 25% of the overall budget, followed by medical personnel (20%) and administration (12%). The cost per DALY averted in the base-case scenario was $1,128, which is 76% of the gross domestic product per capita, demonstrating that treating children with cancer in Haiti is very cost-effective according to the World Health Organisation Choosing Interventions that are Cost-Effective (WHO-CHOICE) threshold. In the most conservative scenario, the cost per DALY averted was cost-effective by WHO-CHOICE criteria. Our data will add to the growing body of literature illustrating a positive return on investment associated with diagnosing and treating children with cancer in even the most resource-limited environments. We anticipate that these data will aid local stakeholders and policymakers when identifying cancer control priorities and making budgetary decisions.

## Background

Globally, an estimated 400,000 children younger than 15 years develop cancer, of whom approximately 43% are never diagnosed [1, 2]. Over 90% of these children come from low- and middle-income countries (LMICs), where the cancer 5-year net survival can range from 10% to 60% [3], rather than the over-80% observed survival in high-income countries [4]. Those diagnosed with childhood cancer in LMICs experience not only a significant survival gap, but also an annual 9.4 million disability-adjusted life years (DALYs), a composite measure of excess mortality and excess morbidity [5]. Thus, in LMIC settings, childhood cancer ranks as a major contributor to both cancer and childhood-related disease burden globally.

Despite the scope of the problem, the assumption that childhood cancers are too expensive to include in essential health packages has gone unchallenged until recently [4, 6]. Skepticism about scaling up access to treatment, early detection and even palliation for children with incurable disease in LMICs is common. Nonetheless, several recent studies, from predominately middle-income countries, have challenged this notion. A systematic literature review summarised all cost and cost-effectiveness studies addressing paediatric oncology treatment in LMIC settings that were published up to 2019, in which 30 studies were included representing 22 countries [7]. More recently, cost-effectiveness analyses were conducted to assess treating paediatric oncology patients in six African countries [8–10]. Results from this growing body of literature suggest that treating paediatric cancer patients in upper and lower-middle income countries is often achievable and cost-effective [7–10] yet only a small number of studies have evaluated these outcomes in low-income countries (LICs).

Haiti is a Caribbean LIC in which little has been published regarding the incidence, treatment or cost of treating paediatric cancers. According to the World Bank, Haiti remains the poorest country in the Latin America and Caribbean region and among the poorest countries in the world [11]. In 2018, Haiti had a population of 11,123,183, a gross domestic product (GDP) per capita of $1,479 United States dollars (USD), life expectancy of 64 years, and a human development index ranking of 170 out of 189 countries [11]. Nos Petit Frères et Sœurs-St. Damien Hospital (NPFS-SDH) is the only paediatric hospital and has the only dedicated paediatric oncology department in Haiti; it admits an average of 60–75 patients with newly diagnosed paediatric cancer annually.

In this study, our objective was to assess the cost and cost-effectiveness of the treatment of all types of childhood cancers at a paediatric cancer unit in Haiti by collaborating with NPFS-SDH in Tabarre, Haiti. These findings will add to the growing body of literature illustrating the ability to diagnose and treat paediatric cancer patients in the lowest income settings. Additionally, these data may aid policymakers when prioritising their essential health benefits.

## Methods

The same methodological approach first introduced by Fuentes-Alabi *et al* [12] to examine the cost and cost-effectiveness of paediatric cancer treatment in El Salvador was applied. The initial step was to convene critical stakeholders to identify services available. For consistency, a modified abstraction tool applied by prior publication was used to collect the data with the cost categories and assumptions to calculate the cost-effectiveness [8, 12, 13]. Because NPFS-SDH is the only centre that offers paediatric oncology service in the country, we made a simplifying assumption that data from the hospital reflects national-level statistics. Ethics approval was not required as all data were administrative or based on existing outcomes (survival); no primary patient information was collected.

### Study setting

NPFS-SDH was founded as a Catholic relief charity to serve as a paediatric hospice facility for children dying of HIV/AIDS. The hospital later developed into a full-service children’s hospital housing the only paediatric oncology program in the country [14]. Situated in metropolitan Port-au-Prince, NPFS-SDH has 228 beds and treats nearly 19,000 patients per year, 0.3% of whom have cancer. Approximately 60–75 new cases of cancer among patients younger than 14 years are diagnosed annually at NPFS-SDH. To care for these children, the hospital has dedicated 13 inpatient beds and has space for an active outpatient clinic. In 2017, approximately 30% of patients received a diagnosis of leukaemia or lymphoma, and 70%, solid tumours. Paediatric cancer patients are cared for by a multidisciplinary core team of two oncology paediatricians, a general paediatrician, residents and medical students on rotations, registered nurses, a psychologist and a social worker. Due to the lack of additional paediatric oncology services in Haiti, NPFS-SDH admits patients from all ten geographical territories in the country. The paediatric oncology program is primarily financed by NPFS-SDH; however, St. Jude Children’s Research Hospital funds staff salaries, chemotherapy and other essential medications and access to radiotherapy in the Dominican Republic.

### Data collection

All data were collected from the hospital records for either year 2017 or 2018, based on availability. A healthcare costing perspective was adopted that was inclusive of all costs regardless of the source of funding (e.g., government, philanthropic entities and out-of-pocket). Conversion from Haitian Gourdes to USD was carried out using the average exchange rate between January and December 2017.

The data collection tool categorised costs into 11 main categories: 1) medical; 2) non-medical personnel; 3) housing, including room and board during inpatient care, intensive care unit (ICU) and short-term patient and family accommodation near the hospital when needed; 4) outpatient visits, including consult visits, ambulatory chemotherapy visits, bone marrow aspirations and biopsies and intrathecal chemotherapy; 5) imaging, including X-ray, computer tomography and ultrasonography; 6) laboratory services; 7) medications (chemotherapy and supportive medications); 8) blood bank services; 9) radiotherapy; 10) surgery; and 11) central administration costs, such as utilities, purchasing and contracting services.

Personnel costs were calculated by multiplying the number of employees in each position by their respective annual salary and the self-reported proportion of their time dedicated to the paediatric oncology unit. This information was obtained from the human resources director for the entire hospital, for the year 2018. Data included both medical staff, such as physicians, pharmacists and pathologists, and non-medical personnel, such as human resources staff, social workers, psychologists, data entry personnel and housekeeping staff.

Housing and outpatient visit cost categories were calculated by multiplying the annual frequency of inpatient nights, outpatient visits and ambulatory chemotherapy visits retrieved from the hospital records by the unit cost obtained from the World Health Organisation–Choosing Interventions that are Cost-Effective (WHO-CHOICE) [15] costing tool for 2008. Costs from the tool were inflated to 2017 by applying country-specific annual GDP deflator index growth rate, published by the World Bank [16]. The unit cost for a day spent in the ICU was assumed to be three times that of an inpatient stay [17]. The local housing unit cost was based on the average cost of a hostel night. The cost of bone marrow aspirations and intrathecal chemotherapy were provided as a bulk annual cost for year 2018.

Regarding diagnosis- and prognosis-related cost categories, laboratory costs were inclusive of routine laboratory tests as well as pathology. For the latter, diagnostic consumables costing data were collected partly from NPFS-SDH and partly from St. Jude Children’s Research Hospital, where some samples were sent for analysis. Imaging was costed from NPFS-SDH for year 2017.

Chemotherapy costs were calculated by retrieving NPFS-SDH’s archival records of all medications purchased in year 2017. Costs associated with supportive medications were calculated based on the 9-month consumption rate of NPFS-SDH in 2017, then multiplied by a factor to estimate the total annual figure. Over 240 supportive medications were considered, including antibiotics, antivirals, corticosteroids and vitamins.

Patients who needed radiation therapy were sent to the Dominican Republic because such facilities were not available in Haiti. Therefore, the cost of the round trip, accommodation and visas for the patient and their parent had to be included in addition to the radiation therapy costs. Actual costing data of the 14 patients sent in 2017 and the 13 sent in 2018 were collected from NPFS-SDH to estimate the cost per patient. Surgical costs were also retrieved from NPFS-SDH for 9 months and extrapolated to calculate the annual total. Costs associated with the blood bank were estimated based on blood and platelet units in addition to cross-matching and blood grouping tests. Central administration costs were calculated based on the prorated proportion of 12% of the total cost of the paediatric cancer unit, in alignment with prior approaches [8, 12, 13].

### Cost-effectiveness analysis

Cost-effectiveness calculations were conducted in alignment with the WHO-CHOICE recommendations [18]. Specifically, interventions costing less than the GDP per capita for each DALY averted are considered ‘very cost-effective’ and those costing less than triple the GDP per capita per DALY averted are considered ‘cost-effective’.

In the model, we assumed that all children with diagnosed cancer would die if not treated. The cost per life saved was calculated by combining the cost of each new diagnosis with the estimated 5-year survival rate (Table 1 summarises the key parameters). An overall 35% 5-year survival rate was calculated for all types of cancers based on hospital data. The cost per life saved was then converted to cost per DALYs averted using Haiti’s life expectancy of 64 years, with the mean age at diagnosis being 6 years. Although the length of treatment of individual childhood cancers varies, we used 1 year as the median duration of therapy. During treatment, children experience diminished quality of life; we accounted for this by using the global burden of disease disability weight of 0.288 [19].

Twelve scenarios were conducted altogether. For the base case, normal life expectancy was assumed with no utility adjustment for excess morbidities associated with therapy-related late-effect. To be conservative, three additional scenarios were explored with utilities adjusted to account for late-effect morbidities. One of the three scenarios assumed normal life expectancy and the other two assumed a 15% or 30% reduction in life expectancy, respectively. For each of the four scenarios, we applied a base case discount rate of 3%, as recommended by the WHO-CHOICE guidelines [18]. Additional estimates using 0% and 6% were also calculated as sensitivity analyses.

## Results

### Costs

**Figure 1.**
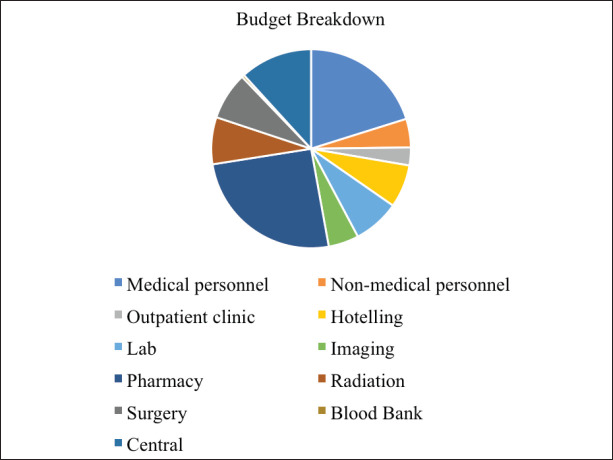
Budget breakdown for each of the 11 cost categories.

Seventy-four patients with newly diagnosed cancer were admitted to NPFS-SDH hospital in 2017. The total cost required to run the paediatric oncology treatment unit for that year was calculated as $803,184, meaning $10,854 per newly diagnosed patient. The breakdown of the cost categories and the cost of each category is presented in Table 2 and Figure 1. The single largest cost category was pharmacy, with 25% of the overall budget, followed by medical personnel, which accounted for 20%.

### Cost-effectiveness analysis

Table 3 presents the results of the base case and sensitivity analysis scenarios of the cost-effectiveness calculations. The cost per DALY averted was $1,128 in the base case, which assumed a 3% discounting rate, normal life expectancy, and no utility adjustment for late-effect morbidity. Sensitivity analyses with varying assumptions regarding the discounting rate, life expectancy and late-effect morbidity revealed a cost per DALY averted of $537–$2,087, which translates into 36%–141% of the country’s GDP per capita of $1,479. Thus, even in the most conservative scenario, the cost per DALY averted was cost-effective by WHO-CHOICE standards. The annual years saved due to treatment were 712 years on the country level, in the base case scenario.

## Discussion

Ours is the first study to assess the cost and cost-effectiveness of treating childhood cancer in Haiti. Our results showed that, even in the most conservative scenario, treating childhood cancer was cost-effective and potentially very cost-effective according to the WHO-CHOICE thresholds, in Haiti, an LIC with one of the lowest human development index rankings in the world. This is particularly impressive given our long-term survivorship assumptions regarding the anticipated reduction in life expectancy are likely pessimistic as they were based on data from the United States, where patients receive more intensive treatment and thus experience higher toxicity- and treatment-related mortality.

All paediatric oncology treatment cost-effectiveness studies conducted in LMICs and published from inception to 2019 were recently summarised in a systematic literature review by Fung *et al* [7]. Since this publication, four additional studies were published, to our knowledge. Githang’a *et al* [8] followed a similar methodological approach to ours and analysed the cost-effectiveness of treating childhood cancers in four countries in Sub-Saharan Africa; Kenya, Tanzania, Nigeria and Zimbabwe [8]. Soliman *et al* [10] based the analysis on real-world data from Egypt, Genemo *et al* [20] used real-world data from Ethiopia and Kiros *et al* [9] employed a decision analytical model and used data from a different hospital in Ethiopia. Our findings are consistent with these previous studies, which concluded that treating childhood cancer is cost-effective or very cost-effective according to the WHO-CHOICE cost-effectiveness threshold [7–10]. Moreover, the ratio of the cost per DALY averted to the national GDP per capita in our base case was 76%, consistent with the 9%–80% range reported in Fung *et al* [7] systematic literature review. Additionally, the breakdown of the budget among different cost categories was also in line with previous findings, which showed that personnel, chemotherapy, surgery and administration usually ranked the highest among other cost categories [7].

Allocation of resources among different cost categories in our study were comparable with that of three previous publications [8, 12, 13] encompassing El Salvador, Ghana, Kenya, Tanzania, Nigeria and Zimbabwe, six LMICs where all types of paediatric malignancies were included and cost categories were classified in the same manner (Table 4). Other studies summarised in the literature review, could not be included in this comparison because they only focused on one or two types of malignancies, while studies from Egypt and Ethiopia, classified cost categories differently, which would have led to a misleading comparison and interpretation of findings. Among the countries included in Table 4, Haiti had the lowest budget share allocated to non-medical personnel and the highest budget share allocated to imaging and radiation. The high cost of radiation for those in Haiti could be explained by the need to send those patients to the Dominican Republic to receive the therapy, which leads to the additional cost of transportation, visas and accommodation for the patient and their parents.

Of the 30 studies included in Fung *et al* [7] systematic literature review, 22 countries were represented, of which 7 were LICs. None of the LIC analyses included comprehensive analyses of all cancers. Only the studies from El Salvador and Ghana, both LMICs, were comprehensive in their reporting [12, 13] while the remaining 28 were focused on one or two types of cancer. Despite the large proportion of the budget usually dedicated to personnel, patient accommodations, and administration, at least half of the studies did not report these categories. Moreover, 25 of the 30 studies did not conduct any sensitivity analysis, and 27 did not adjust their results for long-term morbidity and early mortality due to cancer-related or treatment-related late effects. Our study adds value by carefully integrating all the categories that are frequently left out, being inclusive of all types of cancers, adopting a healthcare perspective regardless of the funding stream, incorporating several sensitivity analyses, accounting for long-term morbidity and early mortality, and analysing this is an LIC with a high level of deprivation and scarce published data.

Notably, cost-effectiveness does not always mean affordable, especially when the payment is primarily out-of-pocket. A prior study examining the out-of-pocket expenditure of breast cancer patients in Haiti concluded that the median was 60% of potential individual income and could account for more than 91% of the annual earnings of three-quarters of Haiti’s population [21]. Interestingly, patients in this study received treatment free of charge, and the out-of-pocket expenditure was only to cover food, lodging, transportation, childcare, lost wages and other expenses. This raises a broader issue of financial hardship and the ability to access treatment even if it is provided for free. To address material, behaviour and psychosocial toxicities associated with medical costs, governments should take a holistic approach by not only budgeting for treatment costs but also creating social welfare programs and payment plans to provide financial support on the family-unit level.

In terms of health service delivery, nearly half the children who develop cancer die without ever receiving a diagnosis [2] worldwide. Thus, the actual number of patients in Haiti is projected to be considerably higher than the 74 patients per year analysed in this study. Accordingly, scale-up strategies are necessary to diagnose and treat these additional patients. Although no effective interventions for the early diagnosis of children have been reported [22], several work streams could be prioritised such as conducting targeted awareness campaigns, improving referral pathways, mitigating financial hardships, establishing follow-up mechanisms to minimise abandonment and capacity building on both the facility and the personnel levels. Additionally, diagnosing more patients will be associated with higher upfront costs, to establish facilities, train and hire personnel and procure additional medicines and supplies. Therefore, a budget impact analysis should follow this cost-effectiveness analysis to estimate the investment needed, to realistically scale-up paediatric oncology services in Haiti.

Finally, to achieve the WHO Global Initiative for Childhood Cancer goal of raising the global survival rate for paediatric oncology patients to at least 60% by 2030 [23], governments must revisit priorities and mobilise resources not only in middle- income countries, but also in LICs such as Haiti. Historically, a large portion of the resources funding childhood cancer treatment has stemmed from national and international philanthropic sources, which, while helpful, is not always sustainable and may be dissociated from the local context. This continues to occur despite evidence that the medium- and long-term return on investment of treating paediatric oncology is high, with an approximate net return of $3 for each $1 invested, due to the number of productive life years gained when a child’s disability or early death is averted [4], which in turn will result in societal gain and will reflect positively on the country’s GDP. Thus, scientifically sound costing and cost-effectiveness analyses such as ours are powerful tools for the medical community to move this agenda forward on a governmental level and to advocate for equitable access to treatment for children with cancer.

Despite our best efforts, this study has several limitations. First, due to a scarcity of published costing data, we could not adopt a societal perspective that would have accounted for the cost categories mentioned in the previous paragraph. Instead, to avoid making several assumptions, we opted for a healthcare perspective, which was more feasible and reliable. Nonetheless, the framework and template we used in our calculations are flexible and could easily be updated once more data becomes available. Second, we used the WHO-CHOICE cost-effectiveness threshold of one and three times the GDP per capita to indicate treatments that are very cost-effective and cost-effective, respectively. We chose this approach to be consistent with the previous publications and because consensus regarding cost-effectiveness threshold in LMICs is lacking. However, a more conservative opportunity-cost–based cost-effectiveness threshold has been estimated at approximately half the GDP per capita [24]. If this threshold is to be considered, our base case result would switch from very cost-effective to not cost-effective.

## Conclusion

The annual cost of operating a paediatric oncology unit in Haiti that treated 74 patients with newly diagnosed cancer was $803,184 overall or $10,854 per patient. The cost per DALY averted was $1,128, equivalent to 76% of the GDP per capita, demonstrating that treating children with cancer in Haiti is very cost-effective. We anticipate these results will help policymakers in Haiti and other LICs while making budget allocation and prioritisation decisions.

## Conflicts of interest

The authors declare that they have no conflict of interest.

## Funding

NSB, MB, PF and NB are partly funded by The American Lebanese Syrian Associated Charities. The funding source had no role in the design and conduct of the study; management, analysis and interpretation of the data; preparation, review or approval of the manuscript; and decision to submit the manuscript for publication.

## Figures and Tables

**Table 1.** Variables and sources included in the cost-effectiveness model.

Variable	Value	Source
Discount rate	0.03 (0, 0.06)	WHO-CHOICE
Haiti life expectancy, year	64	World Bank [11]
Mean age at diagnosis, year	6	Assumption
Duration of disability (length of therapy), year	1	Assumption
Disability weight during therapy	0.288	Murray and Lopez [19]
Utility score at age 24 year using MEPS	0.826	Yeh *et al* [25]
Utility score at age 35 year using MEPS	0.81	Yeh *et al* [25]
Utility score at age 45 year using MEPS	0.791	Yeh *et al* [25]
Number of new incident cases per year	74	Hospital data for year 2017
Proportion of patients with 5-year survival	0.35	Hospital data for year 2017
Haiti GDP per capita, USD	$1,479	World Bank [11]

WHO-CHOICE: World Health Organisation Choosing Interventions that are cost-effective; GBD: global burden of disease; MEPS: medical expenditure panel survey; GDP: gross domestic product; USD: United States dollar

**Table 2.** Annual costs of operating a paediatric oncology department in Haiti.

Cost category	Cost (USD)	Cost per patient (USD)	%
Medical personnel	$161,942	$2,188	20%
Non-medical personnel	$37,193	$503	5%
Housing	$55,980	$756	7%
Outpatient clinic	$23,292	4315	3%
Pharmacy	$203,609	$2,751	25%
Labs	$60,669	$820	8%
Imaging	$39,619	$535	5%
Radiation	$61,123	$826	8%
Surgery	$61,921	$837	8%
Blood bank	$3,365	$45	0%
Subtotal	$708,713	$9,577	
Central administration	$94,471	$1,277	12%
**Total**	**$803,184**	**$10,854**	

Conversion rate for year 2017: 1 USD = 63.78 Haitian Gourde

**Table 3.** Cost per DALY averted: base case and sensitivity analysis.

Life expectancy (LE)^a^ and late-effect morbidity scenario	Discounting		
	0%	3%	6%
Base case (Normal LE, no utility adjustment for late-effect morbidity)	$537^b^	$1,128^b^	$1,920^c^
Normal LE + utility adjustment for late effect morbidity	$567^b^	$1,151^b^	$1,932^c^
15% Reduction in LE + utility adjustment for late-effect morbidity	$678^b^	$1,243^b^	$1,986^c^
30% Reduction in LE + utility adjustment for late-effect morbidity	$845^b^	$1,388^b^	$2,087^c^

a Decrements in life expectancy selected based on Yeh *et al* [25]

b Very cost-effective

c Cost-effective

**Table 4.** Comparison of childhood cancer care costs in other countries^a^ to those in Haiti^b^.

Cost category	Ghana (13)	El Salvador (12)	Kenya (8)	Tanzania (8)	Nigeria (8)	Zimbabwe (8)	Haiti
Medical personnel	34.6%	16.2%	17.70%	35.8%	43.0%	28.9%	20.2%
Nonmedical personnel	11.5%	5.4%	5.9%	11.9%	14.3%	9.6%	4.6%
Housing	5.8%	4.5%	15.6%	5.7%	1.3%	2.7%	7.0%
Outpatient clinic	Not reported	2.6%	0.0%	0.0%	0.0%	10.8%	2.9%
Pharmacy	7.9%	31.8%	38.8%	19.4%	7.8%	29.2%	25.4%
Pathology	2.7%	11.5%	3.6%	4.8%	4.3%	1.7%	7.6%
Imaging	1.9%	1.4%	1.6%	2.1%	0.1%	2.1%	4.9%
Radiation	0.9%	1.0%	1.3%	2.3%	0.0%	0.0%	7.6%
Surgery	22.7%	2.5%	2.8%	5.1%	16.3%	2.7%	7.7%
Blood services	0.2%	9.8%	1.0%	0.1%	1.2%	0.3%	0.4%
Central administration	11.8%	11.9%	11.8%	11.8%	11.8%	11.8%	11.8%
Mean cost/year per child with newly diagnosed cancer (USD)	$9,781	$28,707	$31,344	$2,453	$5,727	$2,338	$10,854
Total annual cost (USD)	$1,662,771	$5,195,800	$4,012,059	$360,558	$229,076	$364,706	$803,184

a Data from previous studies

b Data from the current study
